# Super resolution for root imaging

**DOI:** 10.1002/aps3.11374

**Published:** 2020-07-30

**Authors:** Jose F. Ruiz‐Munoz, Jyothier K. Nimmagadda, Tyler G. Dowd, James E. Baciak, Alina Zare

**Affiliations:** ^1^ Department of Electrical and Computer Engineering University of Florida Gainesville Florida USA; ^2^ Department of Material Sciences and Engineering University of Florida Gainesville Florida USA; ^3^ Donald Danforth Plant Science Center St. Louis Missouri USA

**Keywords:** convolutional neural networks, generative adversarial networks, plant phenotyping, root phenotyping, super resolution

## Abstract

**Premise:**

High‐resolution cameras are very helpful for plant phenotyping as their images enable tasks such as target vs. background discrimination and the measurement and analysis of fine above‐ground plant attributes. However, the acquisition of high‐resolution images of plant roots is more challenging than above‐ground data collection. An effective super‐resolution (SR) algorithm is therefore needed for overcoming the resolution limitations of sensors, reducing storage space requirements, and boosting the performance of subsequent analyses.

**Methods:**

We propose an SR framework for enhancing images of plant roots using convolutional neural networks. We compare three alternatives for training the SR model: (i) training with non‐plant‐root images, (ii) training with plant‐root images, and (iii) pretraining the model with non‐plant‐root images and fine‐tuning with plant‐root images. The architectures of the SR models were based on two state‐of‐the‐art deep learning approaches: a fast SR convolutional neural network and an SR generative adversarial network.

**Results:**

In our experiments, we observed that the SR models improved the quality of low‐resolution images of plant roots in an unseen data set in terms of the signal‐to‐noise ratio. We used a collection of publicly available data sets to demonstrate that the SR models outperform the basic bicubic interpolation, even when trained with non‐root data sets.

**Discussion:**

The incorporation of a deep learning–based SR model in the imaging process enhances the quality of low‐resolution images of plant roots. We demonstrate that SR preprocessing boosts the performance of a machine learning system trained to separate plant roots from their background. Our segmentation experiments also show that high performance on this task can be achieved independently of the signal‐to‐noise ratio. We therefore conclude that the quality of the image enhancement depends on the desired application.

Over the past decade, advances in sensing devices and computer systems have allowed for the proliferation of high‐throughput plant phenotyping systems (Das Choudhury et al., 2019). These systems are designed to acquire and analyze a large number of plant traits (Han et al., 2014; Krieger, 2014), including the measure of small structures, such as the venation network of leaves (Endler, 1998; Green et al., 2014). However, the characterization of plant roots is more challenging because they are “hidden” in the soil (Atkinson et al., 2019), which limits the type of sensors and techniques that can be applied.

A number of types of methods have previously been used to analyze root traits. Non‐imaging‐based in situ methods estimate the traits of the root system architecture (RSA) based on their correlations with chemical or physical properties. For example, Dalton (1995) and Cseresnyés et al. (2018) used the plant root electrical capacitance to estimate the root mass, modeling the RSA as a resistance‐capacitance circuit. Likewise, Cao et al. (2011) employed an electrical impedance spectroscopy approach to model the RSA based on the frequency response. The disadvantage of these methods is that they provide a simplified description of the RSA and thus do not provide morphological details.

Other researchers have used destructive methods, in which the RSA is destroyed during or after the imaging process. The most basic of this type is “shovelomics,” which consists of washing out the roots of the soil (Trachsel et al., 2011). Shovelomics can be applied to plants grown in any type of soil, in contrast with other root phenotyping techniques that are limited by the physical properties of the environment. It is not ideal for high‐throughput work, however, because the manual excavation of the roots is labor‐intensive and tedious. Furthermore, most thin roots are lost in this process.

Another category of root phenotyping method is imaging under controlled conditions. Roots can be observed using rhizotrons, structures with windows that contain the soil in which the plants are grown (Taylor et al., 1990). Alternatively, 3D imaging of the RSA can be carried out on plants grown in special substrates, such as transparent substrates or easy‐to‐remove types of soil (Clark et al., 2011). These procedures allow the acquisition of high‐quality images, but their main disadvantage is that the imaging is not performed in situ, meaning the knowledge that can be inferred using them is limited.

Root phenotyping has also been performed using intrusive methods, in which the acquisition device is introduced into the ground. In this category, we include the minirhizotrons that use a camera fixed into the soil through a tube to record sequences of pictures of parts of the RSA (Johnson et al., 2001), as well as soil coring (Wu et al., 2018). Although these methods do not necessarily result in the destruction of the RSA, they disturb the roots and soil, which might affect the natural root–soil interactions (Kolb et al., 2017). The disturbance can be worse when the devices are introduced and extracted frequently, or when they are installed in difficult substrates such as stony soils (Majdi, 1996).

In contrast, some researchers use non‐intrusive methods to study RSAs in situ, without disturbing the roots or the soil. Barton and Montagu (2004) tested the use of ground‐penetrating radar for this purpose, revealing that it was possible to detect tree roots 1 cm in diameter buried in the soil at a depth of 50 cm; unfortunately, this technology is currently limited to the detection of the roots of trees or woody plants (Hirano et al., 2009; Araus and Cairns, 2014). X‐ray computed tomography (Tabb et al., 2018) and magnetic resonance imaging (MRI) (Pflugfelder et al., 2017) technologies, which involve scanning using devices traditionally used for medical applications, can be grouped into the non‐intrusive category if the complete plant can be scanned in the device (e.g., plants grown in pots). On the other hand, X‐ray computed tomography and MRI are considered intrusive techniques when used to scan washed root systems or soil cores for RSAs removed from the field. In addition to these available approaches, more are currently being developed, including backscatter radiography (Cui et al., 2017).

The root system is responsible for water and nutrient absorption, and it is the first barrier to the changing environment. It affects many seemingly distant processes, such as plant growth, CO_2_ assimilation, and fruit development (Akinnifesi et al., 1998; Chen et al., 2019). The development of high‐throughput root phenotyping methods requiring low labor inputs is crucial for elucidating these systems, which is vital for a wide range of plant research. As mentioned above, the acquisition of high‐resolution (HR) imagery of roots in the field using non‐intrusive methods remains a challenge. An effective super‐resolution (SR) algorithm that complements the imaging process by inferring HR details not clearly delineated by the sensing device is therefore desired for the deployment of these systems in real‐world applications.

The SR problem consists of estimating HR images from low‐resolution (LR) images. SR has been used to overcome hardware limitations in applications that heavily rely on high‐quality images, such as medical diagnosis (Zhang et al., 2012; Zhang and An, 2017). Many SR methods in the literature use mathematical transformations of the original data to learn the LR‐to‐HR mapping (Yang et al., 2010; Zeyde et al., 2012). For instance, methods based on sparse representations reconstruct each image using a weighted combination of *words* from a set of basic patterns called a *dictionary*. A set of LR and HR words are learned from training data, after which an SR image is obtained by replacing the LR dictionary words with HR dictionary words. Recently, data‐driven SR models based on deep learning algorithms with convolutional neural networks (CNNs) have become more popular than the sparse representation‐based models. The SR deep learning algorithms are preferred in many cases because they generally exhibit a better performance, and can be applied as a “black box” when enough training data are available (Wang et al., 2015; Ledig et al., 2017). In particular, SR generative adversarial networks (SRGANs) have shown high performance levels in the estimation of HR detail loss during a degradation process (Ledig et al., 2017). To the best of our knowledge, SR deep learning models for root imagery have not been extensively studied. Additionally, there is no consensus regarding an effective SR performance measure in this context because it has been observed in previous studies that reconstruction accuracy (the pixel‐by‐pixel comparison of an HR–SR pair) and perceptual quality (comparison of the visual features of an HR–SR pair) are not directly correlated (Blau et al., 2019).

Here, to enhance plant root imagery, we adapt two state‐of‐the‐art deep learning approaches, the fast SR convolutional neural network (FSRCNN) proposed by Dong et al. (2016), and the SR generative adversarial network (SRGAN). We train the SR models with LR–HR data from two non‐root data sets (DIV2K and 91‐image) and three plant root data sets (from *Arabidopsis thaliana* (L.) Heynh., wheat [*Triticum aestivum* L.], and barley [*Hordeum vulgare* L.]). These data sets were selected because they contain considerably different textures and shapes, which encourages the model to find a general solution. In addition, to facilitate the training of the generator (the part of the SRGAN that converts LR into HR images), we introduce a modification by implementing multiple discriminators (the part of the SRGAN that evaluates the quality of the SR images). In the loss function (i.e., the part of the model that computes the quality of the estimated parameters), we consider the mean square error between HR and LR (which reduces the reconstruction error, as it is low if the pixel values are similar) and the adversarial loss (which encourages the network to learn to add HR details to the LR image). To evaluate the SR performance, we use two methods: (i) computing the standard signal‐to‐noise ratio (SNR) between the SR image and the original HR image, and (ii) computing the intersection over union (IoU) when applying the SegRoot network (Wang et al., 2019).

## METHODS

### Data sets

In this study, we used five publicly available data sets to train the SR models. We used two non‐plant‐root data sets, DIV2K (https://data.vision.ee.ethz.ch/cvl/DIV2K/ [accessed 11 June 2020]) and 91‐Image (https://www.kaggle.com/ll01dm/t91‐image‐dataset [accessed 11 June 2020]). DIV2K is a data set of natural images that has been used by others to train and test SR algorithms (Timofte et al., 2017). We trained our models on the grayscale version of this training data set (800 images). The 91‐Image information is a classical data set commonly used in SR studies. We also used three plant‐root data sets, including an *A. thaliana* data set for root phenotyping analysis (https://zenodo.org/record/50831#.XjIAPVNKhQI [accessed 11 June 2020]) (Bouché et al., 2016); a data set consisting of 2614 images of wheat seedling roots (http://gigadb.org/dataset/100346 [accessed 11 June 2020]) (Atkinson et al., 2017); and a set of 3D magnetic resonance images of barley roots (https://www.quantitative‐plant.org/dataset/3d‐magnetic‐resonance‐images‐of‐barley‐roots [accessed 21 June 2020]), which also contains WinRHIZO images of the barley roots.

In our experiments, we grouped the three plant‐root data sets into a single data set named “Roots.” Figure 3 shows examples of the plant‐root data sets used for training the SR model. To test the performance of the SR models, we used a data set of 65 soybean (*Glycine max* (L.) Merr.) roots (https://github.com/wtwtwt0330/SegRoot [accessed 11 June 2020]) (Wang et al., 2019).

### SR model training

Many CNN architectures that enable the mapping of LR images into SR images can be found in the machine learning literature. In this study, we used two state‐of‐the‐art CNN‐based models, FSRCNN and SRGAN, to convert LR root images to SR images. FSRCNN is a model that exhibits a similar performance to other state‐of‐the‐art SR techniques, but its execution is considerably faster, making it convenient for comparing different training data sets. Appendix 1 contains a description of the parts of this network. SRGAN is a machine learning system formed by two blocks, a discriminator (*D*) and a generator (*G*). The function of *D* distinguishes between the SR images and real HR images. On the other hand, *G* aims to generate SR images capable of fooling *D*. In Appendix 2, we describe the SRGAN model in detail.

In our experiments, we trained nine SR models: (1) FSRCNN‐DIV2K: FSRCNN trained with the DIV2K data set; (2) FSRCNN‐91‐image: FSRCNN trained with the 91‐image data set; (3) FSRCNN‐roots: FSRCNN trained with the Roots data sets; (4) FSRCNN‐91‐image&roots: FSRCNN‐91‐image model fine‐tuned with the Roots data set; (5) SRGAN‐DIV2K: SRGAN trained with the DIV2K data set; (6) SRGAN‐91‐image: SRGAN trained with the 91‐image data set; (7) SRGAN‐roots: SRGAN trained with the Roots data set; (8) SRGAN‐91‐image&roots: SRGAN‐91‐image model fine‐tuned with the Roots data set; and (9) SRGAN‐MULDIS: SRGAN model trained with three discriminators (one for each data set: DIV2K, 91‐image, and Roots).

For all the SR training experiments, we used a subset of 100 images from the Roots data set as a validation data set, which was used to estimate the performance of the model in terms of the SNR after completing each iteration. After finishing the training process, we identified the parameters that output the highest SNR on the validation test for inclusion in the model. Each model was trained on 100 iterations (the loss function converges with this number of iterations).

### Evaluation

**Figure 1:**
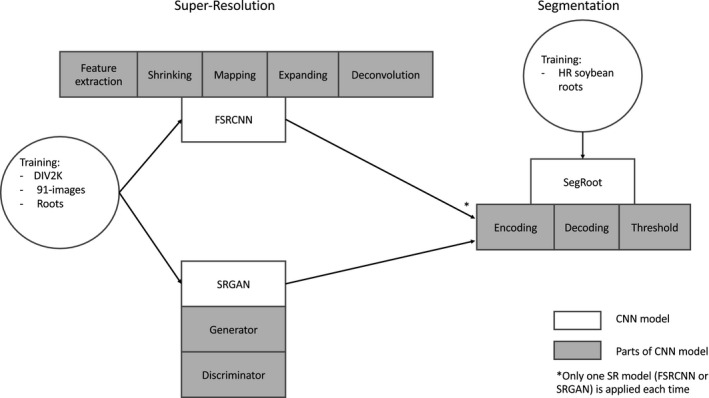
Stages of the super‐resolution (SR) experiments, showing the SR models (FSRCNN and SRGAN) and their constituent parts (left) and the segmentation model, SegRoot (right).

For evaluation purposes, we applied an automatic segmentation on the SR images and quantitatively evaluated the performance of the segmentation. Several U‐net encoder‐decoder architectures have been proposed for the automatic detection and segmentation of plant roots (Xu et al., 2020). In this work, we employed the SegRoot model (Wang et al., 2019). Figure 1 shows the stages of the application of the SR framework to enhance plant root images.

We quantitatively evaluated the SR algorithm performance using two measures: SNR and IoU. SNR is a classic measure for estimating the quality of a recovered signal. It is computed using a pixel‐by‐pixel comparison of the original HR image and the estimated SR image, as follows:

$$SNR=10\log\frac{1}{{∥HR‐SR∥}^{2}}.$$

**Figure 2:**
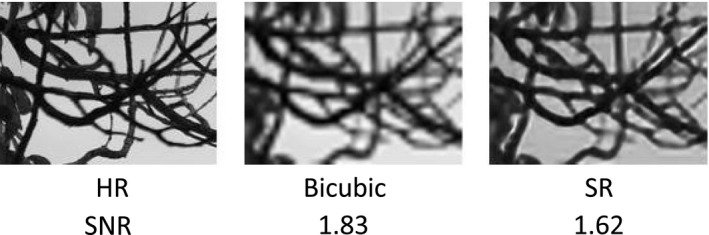
Images demonstrating that the signal‐to‐noise ratio and the visual quality of an image are not always directly correlated.

SNR might not necessarily highlight any enhancement of HR detail, however; for example, in Fig. 2, the SNR (the higher the better) of the image estimated by bicubic interpolation (i.e., increasing the size of the image by interpolating neighbor pixels) was 1.83—higher than the SNR of the SR image (1.62), even though the interpolated image looks blurred. For this reason, we also estimated the effect of applying the SR enhancement as a preprocessing step in an automatic root‐to‐background segmentation process. To this end, we trained the state‐of‐the‐art SegRoot network (Wang et al., 2019) with HR data. We assumed that the segmentation would be more accurate if the input data contained HR details, such as the ones used for training. We compared the binary (‘1’ pixels indicate root, and ‘0’ pixels indicate background) segmented images *B_seg_* with manually labeled images *B_gt_* by calculating the IoU (Rahman and Wang, 2016), also known as the Jaccard Index, as follows:

$$IoU=2\frac{\left|B_{\mathrm{seg}}*B_{\mathrm{gt}}\right|}{\left|B_{\mathrm{seg}}\right|+\left|B_{\mathrm{gt}}\right|}$$

where ‘$\left|\cdot \right|$’ denotes the sum of all the entries of the input matrix and ‘*’ is a pointwise multiplication. IoU values are between 0 and 1 (the higher the better); an IoU value of ‘1’ is when all the target pixels are correctly classified and there are no false positives.

**Figure 3:**
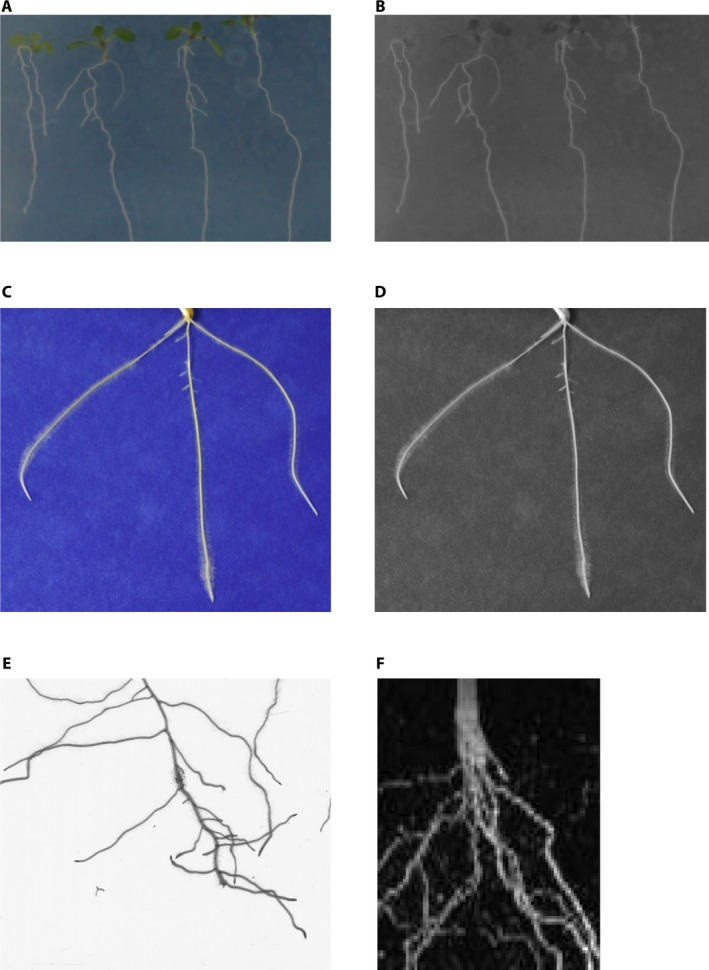
Examples of plant‐root images used to train super‐resolution models. (A, B) *Arabidopsis thaliana* and (C, D) wheat (*Triticum aestivum*) roots shown as RGB (A, C) and grayscale (B, D) images. (E, F) Barley (*Hordeum vulgare*) roots shown as RGB (E) and magnetic resonance (F) images.

## RESULTS

To evaluate the performance of the SR models, we downscaled the images of the soybean data set by a factor of four to reduce their resolution. We used each of the SR models listed above to upscale the test images to their original resolution. We estimated the SNR by comparing the estimated SR images with the original HR images. Next, we used the SegRoot network to automatically classify each pixel in the input image as root or non‐root. As lower and upper bounds, we took the upscaled images by bicubic interpolation, and the original HR images, respectively.

**Figure 4:**
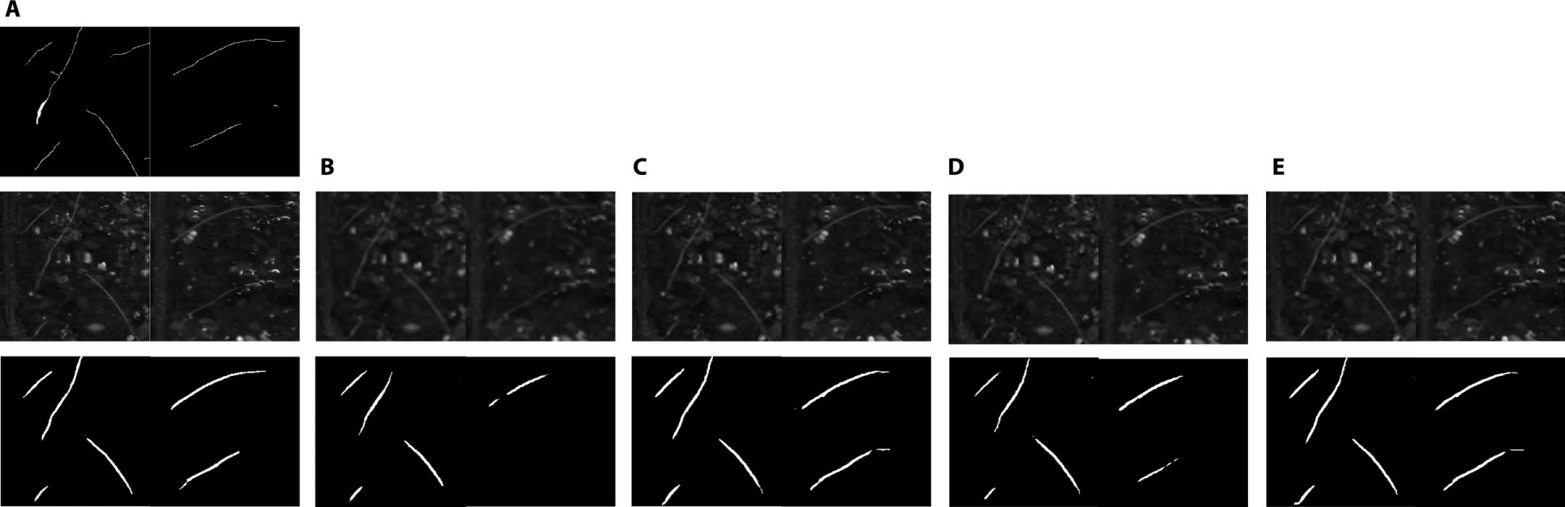
Super‐resolution and segmentation example images (128 × 64‐pixel size) from the soybean (*Glycine max*) data set. From top to bottom: (A) ground‐truth image, high‐resolution (HR) image, and segmentation on the HR image, (B) bicubic image and its segmentation, (C) output of the FSRCNN‐91‐image model and its segmentation, (D) output of the SRGAN‐MULDIS model and its segmentation, and (E) output of the FSRCNN‐91‐image&roots model and its segmentation.

Table 1 contains the SNR and IoU values obtained for the grayscale soybean data set. Segmentation carried out on HR images always exhibited the best performance, likely because the HR details on the images boost the performance of the SegRoot model when analyzing this data. All the SR models outperformed the bicubic interpolation in terms of both SNR and IoU. Regarding only the SR models, three of them (FSRCNN‐91‐image, FSRCNN‐roots, and SRGAN‐MULDIS) exhibited the highest SNR (there is no statistical support for one being better than the others as their standard errors overlap). As two of the three best models use FSRCNN, it might be preferred over SRGAN for this application. There is a mismatch between the SNR and IoU results, however; the model that performs best in terms of the IoU is FSRCNN‐91‐image&roots. Therefore, the features enhanced by the SR models that increase the SNR are not necessarily useful for any given task, such as the applied automatic segmentation. Figure 4 contains examples of SR and segmented images.

**Table 1:** Evaluation of super‐resolution models using a data set of soybean (*Glycine max*) root images. The signal‐to‐noise ratios (SNRs) and intersection over union (IoU) means are presented (standard error in parentheses).^a^

Model	SNR (SE)	IoU (SE)
Bicubic	28.30 (1.37)	0.0984 (0.0098)
FSRCNN‐DIV2K	32.60 (0.19)	0.1313 (0.0106)
FSRCNN‐91‐image	**33.10 (0.20)**	0.1419 (0.0108)
FSRCNN‐roots	**33.05 (0.20)**	0.1623 (0.0111)
FSRCNN‐91‐image&roots	32.48 (0.19)	**0.1709 (0.0110)**
SRGAN‐DIV2K	32.48 (0.19)	0.1402 (0.0106)
SRGAN‐91‐image	32.47 (0.19)	0.1327 (0.0107)
SRGAN‐roots	32.71 (0.19)	0.1485 (0.0108)
SRGAN‐91‐image&roots	32.66 (0.20)	0.1536 (0.0108)
SRGAN‐MULDIS	**33.05 (0.20)**	0.1415 (0.0108)
HR	—	0.2003 (0.0122)

^a^ Grayscale rows are lower and upper bounds (bicubic and high‐resolution, respectively). Boldfaced values correspond to the models that exhibited the highest performance.

The average processing time of a 64 × 64‐pixel image was 0.2248 s using the SRGAN‐based models, 0.2170 s for the FSRCNN models, and 0.0003 s using bicubic interpolation. Note that bicubic interpolation is an upscaling method that does not require training. All the computational experiments were performed on a Linux CentOS 7 machine, x86_64, Intel Xeon CPU @3.60 GHz (Intel, Santa Clara, California, USA) with a GPU GeForce RTX (Nvidia, Santa Clara, California, USA). For the implementation, we used the deep learning framework PyTorch 1.2.0 (Paszke et al., 2019).

## DISCUSSION

We designed a framework for the application of deep learning–based SR models to enhance plant root images. In our experiments, we evaluated the SR models in terms of both the reconstruction capability (by SNR), and the boosting of the images for performing automatic segmentation (by IoU). We demonstrated that the SR models outperform the basic bicubic interpolation even when trained with non‐root data sets. Furthermore, our segmentation experiments showed that a high performance on this task can be achieved independently of an enhanced SNR. We therefore conclude that the quality of the image enhancement depends on the application.

The image processing pipeline could also include other stages, such as denoising and contrast enhancement. To incorporate any new stage, we recommend using the two‐section evaluation method that we applied in this study: evaluate the performance on the processed image directly, and evaluate the results when performing a machine learning task on the processed image. In addition, we suggest that SR models could be used to analyze root system architectures (a stitching method might be needed to put together pieces of SR images) and improve the performance of other machine learning tasks, such as feature extraction and classification.

Future work could include the application of the proposed SR framework to images acquired in the field. Here, we generated LR samples by downscaling the original HR images; therefore, an extension of this work might consider using an alternative to transform HR into LR images, such as “blind SR kernel estimation” methods.

## Data Availability

Five publicly available data sets were used in this study; these are available as follows: DIV2K (https://data.vision.ee.ethz.ch/cvl/DIV2K/), 91‐Image (https://www.kaggle.com/ll01dm/t91‐image‐dataset), *Arabidopsis thaliana* data set (https://zenodo.org/record/50831#.XjIAPVNKhQI), wheat seedling data set (http://gigadb.org/dataset/100346), and barley data set (https://www.quantitative‐plant.org/dataset/3d‐magnetic‐resonance‐images‐of‐barley‐roots). The source code and pre‐trained SR models are available at GitHub and Zenodo (https://github.com/GatorSense/SRrootimaging; https://doi.org/10.5281/zenodo.3940562; Ruiz‐Munoz, 2020).

## APPENDIX 1 Description of the fast super‐resolution convolutional neural network (FSRCNN).

The FSRCNN model is divided into five parts: (1) Feature extraction: FSRCNN consists of a convolutional layer with *d* filters of size 5 × 5 and one input channel. In this case, *d* is considered the LR dimension. This part is denoted by *Conv(5,d,1)*. (2) Shrinking: The purpose of a shrinking layer is reducing the LR dimension. The shrinking procedure is carried out by a convolutional layer of *s* 1 × 1‐filters denoted by *Conv(1,s,d)*, where *s* is smaller than the number of input channels *d*. (3) Mapping: The mapping layer is a non‐linear mapping that aims to estimate a shrunken version of the HR dimension. This layer is implemented as a sequence of *m* 3 × 3 convolutional layers. The number of filters is *s* for each layer. This mapping is denoted by *m*x*Conv(3,s,s)*. (4) Expanding: The expanding layer is implemented using a number, *d*, of SR feature maps estimated by a 1 × 1 convolutional layer (denoted by *Conv(1,d,s)*). (5) Deconvolution: The deconvolution component corresponds to a 9 × 9 deconvolution layer with one filter that upscales *n* times the height‐and‐width input dimensions. The deconvolution components denoted by *DeConv(9,1,d)*.

As suggested by the authors of FSRCNN (Dong et al., 2016), we applied a parametric rectified linear unit (PReLU) after each convolutional layer. Also, we set the parameters *d*, *s*, and *m* as 56, 12, and 4, respectively, which were experimentally demonstrated to be suitable for recovering HR details.

## APPENDIX 2 Description of the super‐resolution generative adversarial network (SRGAN).

A generative adversarial network (GAN) is formed by two blocks, a generator *G* and a discriminator *D*. In this configuration, *G* and *D* play contrary roles, with *G* aimed at generating “realistic‐like fake data” capable of fooling *D*, whereas *D* is continuously trained to identify fake data from real data (Goodfellow et al., 2015). Mathematically, the adversarial setting is formulated as follows

(1) $${\text{min}}_{G}{\text{max}}_{D}E_{x∼p(x)}[\log\left(D(x)\right)]+E_{x∼q(x)}[1‐\log\left(D(G(x))\right)]$$

where E[·] denotes the expectation operator, *x* is a sample (e.g., an image), and *p* and *q* are data distributions (e.g., distributions of LR and HR images). Because this is a min‐max problem, the expression in (1) is both a loss function and a reward function. The optimization problem is solved in an alternating manner. In one step, the loss function is minimized with regard to *G*, such that the output of *G(x)|x~p(x)* is optimized when *D(G(x))* equals 1. On the other hand, the expression in (1) is seen as a reward function that is maximized with regard to *D*. In this case, *D(x)* is a classifier that is trained to output 1 when *x~p(x)*, and 0 when *x~q(x)*.

To choose the architecture of *D*, we need a two‐class classification network, while for *G* we require a network that outputs a matrix of the same size of the input (because the LR image is interpolated to the size of the desired SR image). We evaluated several architectures and selected two for their balance between performance and computational requirements. For the *G* network, we used the convolutional super‐resolution layers of the resolution‐aware convolutional neural network (RACNN) proposed by Cai et al. (2019). For *D*, we designed a two‐class classifier with three convolutional layers and one fully connected layer. For training, we used a batch size of 100, and as an update rule, we applied adaptive moment estimation (Adam), which was also used in the method proposed by Ledig et al. (2017), with a learning rate of 0.001. To create LR training images, we randomly selected 64 × 64‐pixel chunks, downsampled them to 16 × 16 pixels, and upsampled them again to the original size by bicubic interpolation.

In a SRGAN, *x~p(x)* is a sample of a set of LR images, and *x~q(x)* is a sample of a set of HR images. After several iterations, it was expected that *D* would not be able to tell apart HR and SR images, i.e.,* G* learns to convert LR images into SR images very similar to the original HR images. Note that in (1), it was not required for the output of the generator to match the HR version of the LR input, i.e., the content of the generated image might not be the same as in the LR image. To enforce the matching between the HR–LR pairs, we added the squared error between the HR and SR images to the function as follows

(2) $${\text{min}}_{G}{\text{max}}_{D}{\text{E}}_{x,y∼p(x,y)}[\log(D(x))+\log(1‐D(G(y)))]+{∥x‐G(y)∥}^{2}$$

where *x,y~p(x,y)* is a pair of HR (x) and LR (y) images randomly sampled from a set of HR–LR image pairs, and $∥\cdot ∥$ denotes the Euclidean norm. Note that in (2), when minimizing with regard to *G*, two terms are considered, log(1‐D(G(y))) and ${∥x‐G(y)∥}^{2}$, which correspond to the generative adversarial loss and content loss, respectively (Ledig et al., 2017).

We modify the approach described in (2) by incorporating multiple *D_i_* discriminators in the SRGAN architecture (one discriminator per data set); therefore, each discriminator acts as an expert to distinguish HR–SR images on one type of data. The optimization problem is written as follows

(3) $${\text{min}}_{G}{\text{max}}_{D}\;\sum_{i}^{}{\text{E}}_{x,y∼p_{i}(x,y)}[\log(D_{i}(x))+\log(1‐D_{i}(G(y)))]+∥x‐G(y)∥$$

where *D_i_* is the discriminator specialized in the *i*th data set. We hypothesize that, in this way, the generator will output more general SR images because it is more challenging to “cheat” several specialized discriminators (one per type of data) than a general one (a discriminator that distinguishes HR vs. SR images of any kind).
